# New Poly(N-isopropylacrylamide-butylacrylate) Copolymer Biointerfaces and Their Characteristic Influence on Cell Behavior In Vitro

**DOI:** 10.3390/ijms23073988

**Published:** 2022-04-03

**Authors:** Nicoleta-Luminita Dumitrescu, Madalina Icriverzi, Anca Bonciu, Paula Florian, Antoniu Moldovan, Anca Roseanu, Laurentiu Rusen, Valentina Dinca, Florin Grama

**Affiliations:** 1 Lasers Department, National Institute for Lasers, Plasma, and Radiation Physics, 409 Atomistilor Street, 077125 Magurele, Romania; nicoleta.dumitrescu@inflpr.ro (N.-L.D.); antoniu.moldovan@inflpr.ro (A.M.); 2 Ligand-Receptor Interactions Department, Institute of Biochemistry of the Romanian Academy, 060031 Bucharest, Romania; radu_mada@yahoo.co.uk (M.I.); florian_paula@yahoo.com (P.F.); roseanua@gmail.com (A.R.); 3 FOTOPLASMAT Department, National Institute for Lasers, Plasma, and Radiation Physics, 409 Atomistilor Street, 077125 Magurele, Romania; anca.bonciu@inflpr.ro; 4 Faculty of Physics, Biophysics and Medical Physics Department, University of Bucharest, 405 Atomistilor, 077125 Magurele, Romania; 5 Surgical Department, Carol Davila University of Medicine & Pharmacy, 37 Dionisie Lupu Street, 030171 Bucharest, Romania; florin.grama@umfcd.ro

**Keywords:** pNIPAM-co-BA, MAPLE, biointerfaces

## Abstract

Designing and obtaining new synthetic smart biointerfaces with specific and controlled characteristics relevant for applications in biomedical and bioengineering domains represents one of the main challenges in these fields. In this work, Matrix-Assisted Pulsed Laser Evaporation (MAPLE) is used to obtain synthetic biointerfaces of poly(N-isopropyl acrylamide-butyl acrylate) p(NIPAM-BA) copolymer with different characteristics (i.e., roughness, porosity, wettability), and their effect on normal HEK 293 T and murine melanoma B16-F1 cells is studied. For this, the influence of various solvents (chloroform, dimethylsulfoxide, water) and fluence variation (250–450 mJ/cm^2^) on the morphological, roughness, wettability, and physico–chemical characteristics of the coatings are evaluated by atomic force microscopy, scanning electron microscopy, contact angle measurements, Fourier-transform-IR spectroscopy, and X-ray photoelectron spectroscopy. Coatings obtained by the spin coating method are used for reference. No significant alteration in the chemistry of the surfaces is observed for the coatings obtained by both methods. All p(NIPAM-BA) coatings show hydrophilic character, with the exception of those obtained with chloroform at 250 mJ/cm^2^. The surface morphology is shown to depend on both solvent type and laser fluence and it ranges from smooth surfaces to rough and porous ones. Physico–chemical and biological analysis reveal that the MAPLE deposition method with fluences of 350–450 mJ/cm^2^ when using DMSO solvent is more appropriate for bioengineering applications due to the surface characteristics (i.e., pore presence) and to the good compatibility with normal cells and cytotoxicity against melanoma cells.

## 1. Introduction

Despite the progress made in recent decades regarding interdisciplinary research in the development of biomaterials and biointerface design, obtaining materials with controlled characteristics still represents one of the primary key points to be developed and understood in biomedical applications [1,2]. Therefore, designing and tailoring new material biointerface characteristics is correlated to the engineering and manufacturing processes, as well as to the rigorous surface characterization [3,4]. Consequently, it is necessary to identify and incorporate some key interface characteristics into the synthetic materials that can provide appropriate cell-interface or substrate interactions [5]. Currently, the smart polymer layers and interface engineering is a domain of great interest in developing new and efficient biomaterials such as controlled drug delivery vehicles in medical and pharmaceutical applications [6]. Focusing on the ability of the biointerface to influence cell behavior, obtaining coatings with well-defined and controlled characteristics could be an appropriate strategy for biomedical applications. In this context, cell smart bioplatform engineering is a recent field that focuses on the use of thermosensitivity in ‘‘smart’’ drug-delivery platforms/supports, which can react to various external factors such as temperature [7,8], pH [9], light, mechanical perturbations, or magnetic fields [10,11,12]. Poly (N-isopropyl acrylamide) (p(NIPAM) represents one of the most used smart polymers due to its specific characteristics such as pH sensitivity, low toxicity, and thermo-responsive and reversible properties in aqueous solutions [7]. Nowadays, smart polymers, particularly based on pNIPAM, are extensively studied as drug delivery systems for biomedical applications due to their solubility in water, lower critical solution temperature—LCST close to body temperature, and low cell toxicity [13,14]. Several strategies envisage the modulation of specific/particular properties to meet complex demands for pNIPAM-based materials application in the biomedical area (i.e., pH, thermo-response), such as the copolymerization method, which is currently used [10,13]. The copolymerization strategy/technique involves the choice of high biocompatible and non-toxic monomers, which by binding to p(NIPAM), are able to produce copolymeric microgels with new or optimized properties, such as dual responsivity, to fulfill the requirements of a variety of bio-application from molecular switching to drug delivery, where responses to small stimuli changes are relevant [15,16,17,18]. In recent years, temperature- and pH-sensitive copolymers of N-isopropylacrylamide gained a lot of interest in the scientific community as they can be designed for a phase transition near physiological pH or at slightly acidic pH values that fall within acidic gradients found in biology. For example, pNIPAM copolymerized with propylacrylic acid (PAA)) was studied and used as a pH- and temperature-responsive injectable hydrogel system for delivering drugs to regions of local acidosis, as found in wound healing, tumor sites, or sites of ischemia [19,20]. Another monomer used for copolymerization is butyl acrylate (BA), capable of lowering the LCST of the resulted poly (N-isopropyl acrylamide-co-butyl acrylate) p(NIPAM-BA)((C_6_H_11_NO)m(C_7_H_12_O_2_)n) [12,13] at around 24 °C, as compared to homopolymer pNIPAM when prepared by crosslinking (LCST 32 °C). It is also expected to regulate the hydrophilic/hydrophobic balance of p(NIPAM) homopolymer tuning the pH and thermo-responsivity of the resulted p(NIPAM-BA) copolymer microgels in terms of swelling behavior, which contributes to the incorporation/release process of biocompound delivery (i.e., antibiotics, peptides, proteins) and cell attachment [7,10,11,12,21]. One of the biggest benefits of using the p(NIPAM-BA) copolymer is achieving a better interaction with ECM proteins due to the higher hydrophobicity property conferred by the monofunctional BA monomer.

However, the p(NIPAM-BA) copolymer applicability for cell culture was barely studied. Given the fact that the key purpose of using new materials for biological applications, especially for cell culture, is mainly related to starting from two-dimensional (2D) systems (i.e., coatings), which are considered as fundamental research or preliminary exploration. It is essential to have a good understanding of how various cells interact with this material prepared as a coating, and how different surface characteristics in terms of morphology can be obtained and modulated for these applications. Until now, there have only been a few studies related to the biocompatibility of p(NIPAM-BA) copolymer with cells (i.e., 3T3 NIH fibroblasts and human oral epithelial cells) [18,19]. Moreover, the study was done on a macroscopic hydrogel obtained by microemulsion polymerization. Given that the cellular response to an interface is closely related to its structural and architectural characteristics, various methods are envisaged and developed to comply with specific surface or interface requirements of the targeted application. Among the three different strategies for preparation of planar pNIPAM based films (grafting, coating, or both together), Matrix-Assisted Pulsed Laser Evaporation (MAPLE) offers the advantages of tuning and tailoring a wide variety of processing parameters and materials, therefore conferring flexibility for obtaining planar coatings with various characteristics suitable for biomedical applications. In all MAPLE experiments, the material deposited as the coating is first dissolved or suspended in a volatile solvent with a typically low concentration (below five weight percent). The resulting solution is frozen, forming the target to be laser irradiated, and a laser beam is scanned over its surface. The entire process takes place in a vacuum chamber. The very low concentrations used for forming the target allow the laser energy to be mainly absorbed by the solvent, which vaporizes, thereby entangling the solute material and promoting its deposition onto the substrates [22].

One of MAPLE’s unique features is the ability to obtain coatings from a wide range of materials without a structural degradation, containing a one component or multi-component layer and exhibiting controlled features such as thickness and roughness, which are specific interface features [8,20,21,22,23,24,25,26,27].

In this context, we obtain for the first time new poly(N-isopropylacrylamide-butylacrylate) copolymer coatings by using the MAPLE technique, aiming to understand, modulate, and optimize interface characteristics to be used as cell substrates. The obtained nano-biointerfaces based on p(NIPAM-BA) copolymer had different characteristics, from smooth and uniform with low roughness, to grains with nano-spherical shapes such as structures or porous surfaces, with varied pore radius and roughness by changing either the laser fluences used or the target solvent (water, chloroform, and dimethyl sulfoxide (DMSO)). The tailoring of the p(NIPAM-BA) biointerface characteristics (e.g., roughness, pores presence) was correlated with the response in vitro of two cells lines (i.e., normal HEK 293T, murine melanoma B16-F1 cells)**.** The thermo-responsive capacity of the optimized coatings was analyzed by contact angle changes for a temperature shift from 37 to 24 °C, while the hydration capacity was studied by the thickness change during immersion in liquids. The presence of pores, physico–chemical characterization, hydration behavior, and the biological response give the perspective of the p(NIPAM-BA) based coatings to be loaded with bioactive compounds (e.g., antitumoral drugs) for future use as multifunctional and stimuli-responsive platforms targeting the study of tumoral cells.

## 2. Results and Discussions

### 2.1. p(NIPAM-BA) Interfaces’ Morphology Obtained by MAPLE

Physical cues of thermo-responsive surfaces, particularly morphology and topography, have been demonstrated to have remarkable impacts on cell behavior [23]. In our study, the preliminary composition of each matrix (solvent) was analyzed in correlation with the surface chemistry, chemical and morphological characteristics, of the p(NIPAM-BA) final layer to identify the solvent suitable to obtain a uniform, homogeneous coating with an undamaged chemical profile, as well as an appropriate surface characteristic beneficial for cell–biointerface interaction. Since the first use of the MAPLE technique, it was recognized that the solvent plays a fundamental role as a “driving motor” of both the evaporation process and transport of the solute toward the substrate [22]. There are studies, including molecular dynamics simulations of MAPLE processes showing that, depending on the parameters used, a dry, wet, or semidry process can be involved, depending on the parameters used (type of solvent, fluence) and that the solvent might have an active role not only as a solute vector but also in affecting the coating morphology and structure [22,23,24,25]. As evaporation is dependent on the boiling point, our strategy was to use solvents with different freezing/boiling evaporation rates, respectively (T°_Frost_ = 0 °C and T°_Boil_ = 0 °C—water; T°_Frost_ = 19 °C and T°_boil_ = 189 °C—DMSO; T°_Frost_ = −63.5 °C and T°_Boil_ = 61.15 °C—chloroform). The schematic setup involved in obtaining the coatings is shown in Supplementary Figure S1.

In our case, AFM and SEM morphological analysis revealed p(NIPAM-BA) surface characteristics from low roughness to grains with nano-spherical shapes such as structures or porous surfaces, depending on both the fluence and type of solvent used.

#### 2.1.1. Water-Solvent

**Figure 1:**
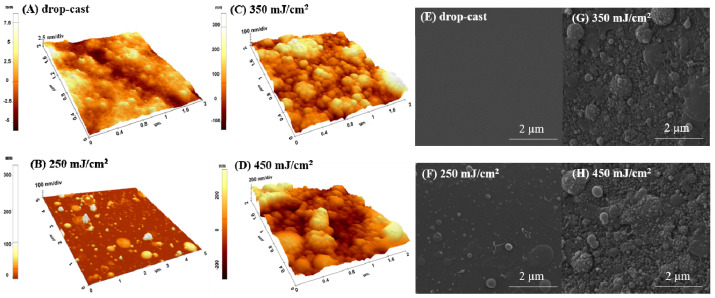
AFM surface images (3D—left) and SEM micrographs (right) of p(NIPAM-BA) layers in water solvent obtained by drop-cast (**A**,**E**) and by MAPLE deposition at 250 mJ /cm^2^ (**B**,**F**), 350 mJ /cm^2^ (**C**,**G**), and 450 mJ/cm^2^ fluences (**D**,**H**). AFM images correspond to areas of 5 × 5 µm^2^ and 2 × 2 µm^2^. SEM magnifications 40,000×.

As shown in Figure 1, by both AFM (Figure 1A–D) and SEM micrographs (Figure 1F,G,H), the coatings are characterized by the presence of nano-globular structures on the surface, with their diameter and density increasing with the fluence. For the drop-cast sample, the presence of grains with nano-spherical shapes can also be observed (Figure 1 A). In the case of the coatings deposited by MAPLE, dimensions are from 10 to 200 nm for the samples transferred at the lower laser fluence of 250 mJ/cm^2^ (Figure 1B,F), while higher laser fluences (350 mJ/cm^2^ and 450 mJ/cm^2^) seem to lead/favor the random agglomeration of the globular grains, whose diameter dimensions exceed 500 nanometers (350 mJ/cm^2^ and 450 mJ/cm^2^) (Figure 1C,D). Regarding the roughness, measured up to 5 × 5 µm^2^, it was found that the values increase with the increasing laser fluence, from 36.3 nm for 250 mJ/cm^2^ to 346 nm for 450 mJ/cm^2^.

The presence of granular and island-like structures formed during MAPLE deposition can be explained based on both MAPLE behavioral deposition and the polar nature of the solvent aspects. It was previously shown that the phase transition’s dependence on the solvent nature (polar or non-polar) and the hydrogen-bond interactions influence the physico–chemical properties, especially morphology [28]. When the material reaches the substrate during evaporation, it initially adheres to its surface as random nano-granular clusters. With the increasing number of pulses, the clusters become more uniform, and the material covers the entire surface. When increasing the fluence, an explanation for the observed agglomeration of the globular grains, whose diameter dimensions exceed 500 nanometers, originates from the mechanisms of target evaporation and ablation causing the polymer and solvent clusters to be ejected towards the substrate [29,30]. This type of rough and inhomogeneous globular morphology is consistent with previous reports of MAPLE films [22,26].

#### 2.1.2. DMSO-Solvent

**Figure 2:**
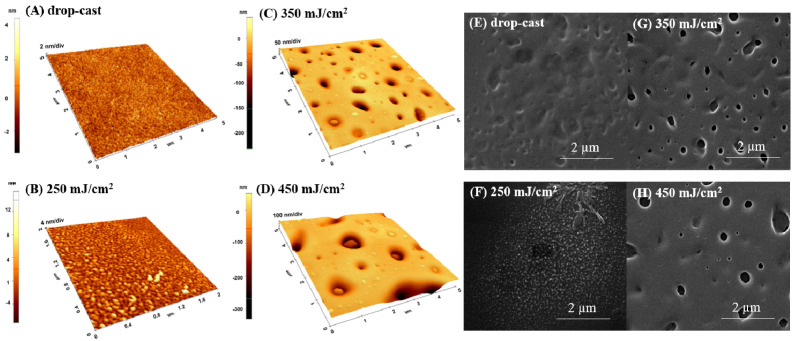
AFM surface images (3D-left) and SEM micrographs (right) on p(NIPAM-BA) coatings when DMSO was used as solvent; (**A**,**E**) drop-cast and MAPLE deposition on Si at 250 mJ/cm^2^ (**B**,**F**), 350 mJ/cm^2^ (**C**,**G**), and 450 mJ/cm^2^ fluences (**D**,**H**). The AFM images correspond to areas of 5 × 5 µm^2^ and 2 × 2 µm^2^; SEM magnifications 40,000×.

In the case of DMSO used as a solvent, the roughness values also increase with fluence (Figure 2) but are lower compared to those obtained for the water solvent (Figure 1). Thus, the surface roughness for drop-cast material was around 0.8 nm and reached 49.4 nm at 450 mJ/cm^2^ fluence (Figure 2D). DMSO favors the formation of pores whose diameter is dependent on the used fluence (Figure 2 left); findings are also confirmed by the SEM images (Figure 2F,G,H—right).

As DMSO has the highest boiling temperature and the lowest evaporation rate, respectively, we can assume that the solvent has an active role as a solute vector, as previously discussed, and this affects the coating morphology [22,24]. The presence of solvent on the substrates during deposition can lead to the formation of a weak H bond between the C-O group of pNIPAM and the methyl group of DMSO, leading to a different surface arrangement [27].

The presence of pores can be beneficial for a wide range of applications, as it has been reported that the increase in pore size facilitates a proper diffusion and adsorption of biologically active compounds and metabolites such as peptides or proteins from the extracellular matrix [13,25,26]. Therefore, the presence of pores and their dimension and distribution, along with the swelling behavior, are essential characteristics for using this type of material to develop future bio-smart cell culture or drug delivery platforms/support.

#### 2.1.3. Chloroform-Solvent

**Figure 3:**
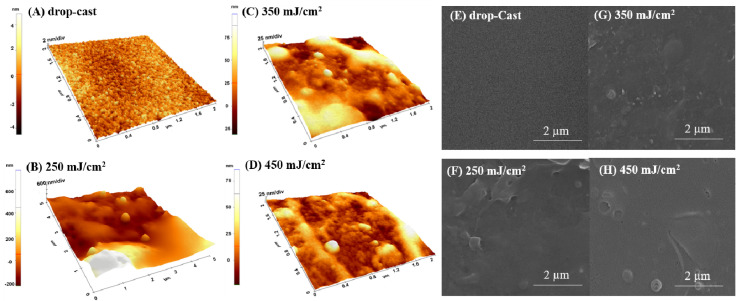
AFM surface images (3D-left) and SEM micrographs (right) on p(NIPAM-BA) coatings when chloroform was used as solvent; (**A**,**E**) drop-cast and MAPLE deposition on Si at 250 mJ/cm^2^ (**B**,**F**), 350 mJ /cm^2^ (**C**,**G**), and 450 mJ/cm^2^ fluences (**D**,**H**). The AFM images correspond to areas of 5 × 5 µm^2^ and 2 × 2 µm^2^. SEM magnifications 40,000×.

The use of chloroform-solvent induced different modifications in the morphology of the p(NIPAM-BA) layers, where a change in the structure’s shape from spherical/droplet-like to elongated ones, due to the melting or fusion of nano-spheres, can be seen (Figure 3). Compared to water (Figure 1), only a few particles with similar shapes are found on the surface of the layers based on p(NIPAM-BA) dissolved in chloroform (Figure 3). Thus, at laser fluence 450 mJ/cm^2^, the morphological and topographic analysis revealed the formation of a blanket-type surface characterized by the presence of spheres or deflated structures with diameter dimensions exceeding 1µm and a higher surface roughness compared to those obtained for lower laser fluences of 250–350 mJ/cm^2^ (Figure 3B,C).

One phenomenon that leads to the appearance of a cluster or droplet-like structures on the surface is related to the fact that, during MAPLE experiments, solvent molecules, together with polymer molecules and intermediate gas-phase solvent molecules, form a cluster that travels towards the substrate through the evaporation. As evaporation takes place from the outside of the cluster, the polymer concentration increases during its movement towards the substrate, where it lands as a nano or microdroplet and leads to the formation of a film with a specific balloon or cluster-like structures on its surface.

Therefore, in the case of chloroform, the evaporation of the solvent leads to the formation of a coating with richer clusters or balloon-like structures, especially when fluences are used, while when using a solvent with a slower evaporation rate, such as DMSO, the pseudo “diffusion” rate of the remaining solvent molecules from the clusters can increase even more. This favors the reorganization on the substrate and the formation of blanket-like porous structures, such as the one shown in the SEM and AFM images.

Altogether, the AFM and SEM studies indicate a linear increase in roughness with increasing laser fluence for all samples deposited by the MAPLE technique (Table 1). Regarding the influence of the solvent, the highest surface roughness was obtained in the case of water, followed by chloroform and DMSO for MAPLE deposition, while for the SC method, the DMSO solvent led to the highest RMS values (Table 1).

### 2.2. p(NIPAM-BA) Interfaces’ Morphology Obtained by Spin Coating (SC)

**Figure 4:**
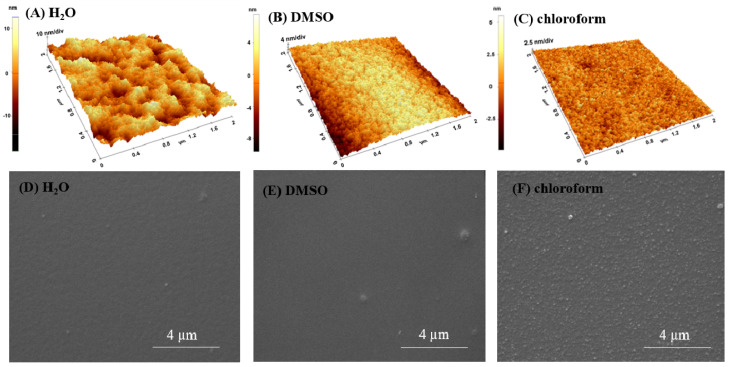
AFM surface images (3D-up) and SEM micrographs (down) on dry layers of p(NIPAM-BA) deposited by spin coating on Si with different rotational parameters (rpm) for each solvent: water, 4500/20 s (**A**,**D**); DMSO × 2 steps (5000/1 min 30 s and 6000/50 s) (**B**,**E**); chloroform, 4500/10 s (**C**,**F**). The AFM images correspond to areas of 5 × 5 µm^2^ and 2 × 2 µm^2^. SEM magnifications 20,000×.

The AFM micrographs of p(NIPAM-BA) layers with three solvents deposited by SC (Figure 4) revealed a completely different morphology than one of the layers obtained by MAPLE but similar to drop-cast layers (Figure 1A,E, Figure 2A,E and Figure 3A,E). All samples are characterized by smooth aspect and low roughness (7 nm for DMSO, 3.6 nm for water, or 1 nm for chloroform, (area of 5 × 5 µm^2^)).

### 2.3. FTIR Investigations of the p(NIPAM-BA) Copolymer Coatings

**Figure 5:**
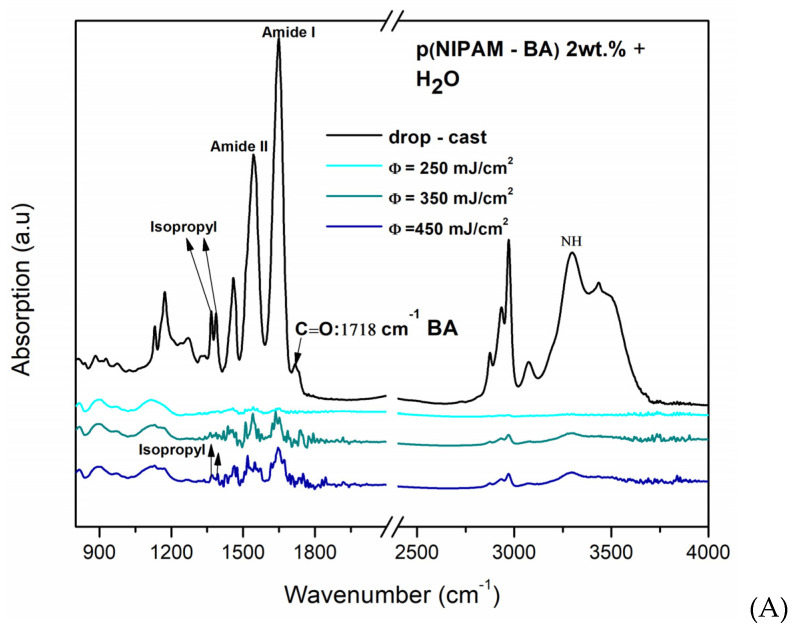

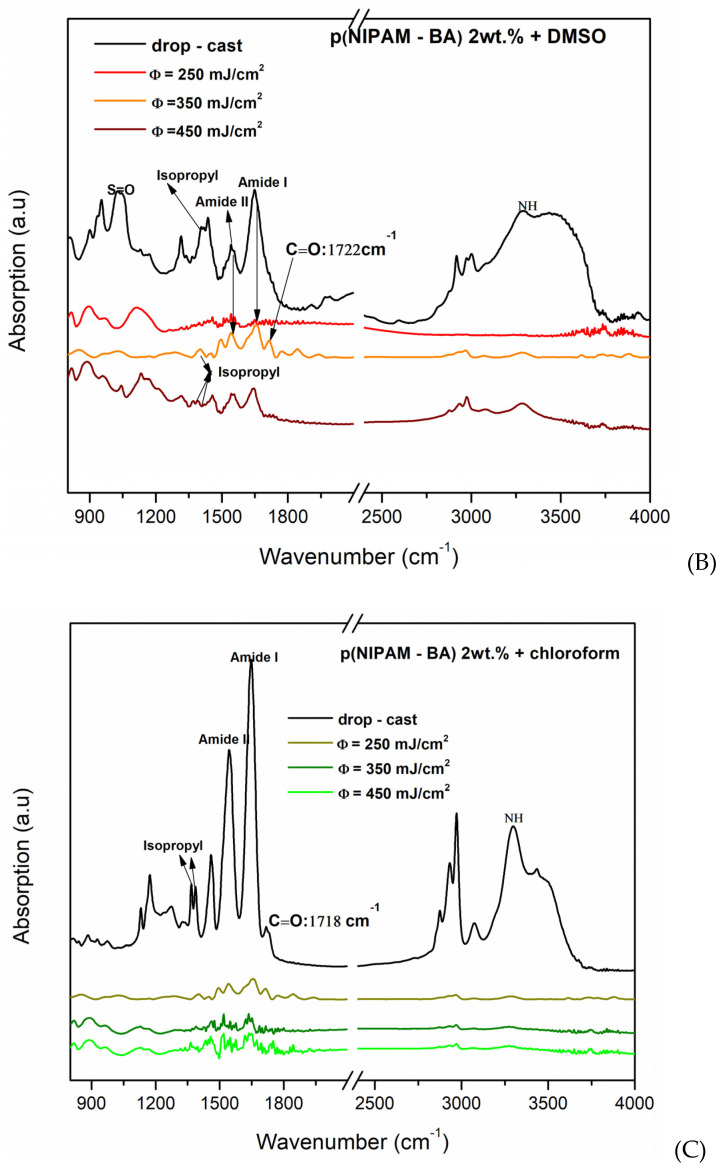
FTIR spectra of drop-cast layer (black line) and MAPLE layers with (**A**) water, (**B**) DMSO, and (**C**) chloroform at varied laser fluence (colored lines).

**Figure 6:**
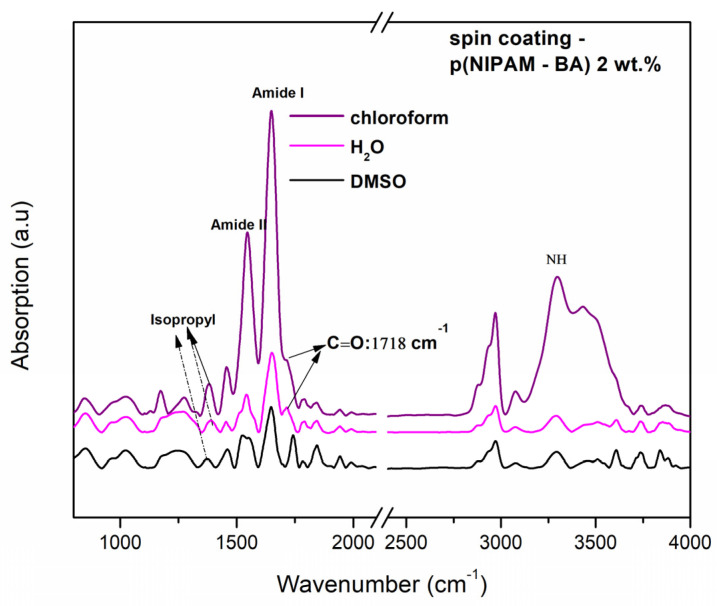
FTIR spectra of spin coated layers with water, DMSO, and chloroform.

The absorption bands characteristic of the p(NIPAM-BA) copolymer deposited by two different techniques and using three solvents (water, DMSO, chloroform) were analyzed by FTIR analysis. The characteristic vibrations of the functional groups present in the p(NIPAM-BA) layers obtained by spin coating (optimized rotational parameters) and MAPLE (250, 350, 450 mJ/cm^2^ laser fluences) depositions using the different solvents were subsequently analyzed and compared with the copolymeric control layers (drop-cast, control/Si) (Figure 5 and Figure 6).

From the above FTIR spectra of the copolymeric coatings obtained by MAPLE (Figure 5) and spin coating (Figure 6), the main characteristic absorption peaks for p(NIPAM) and BA are shown at 3301 cm^−1^ (N–H stretching mode), O-C=O at 1718 cm^−1^, 1649 cm^−1^ (N–C=O, Amide I), and 1543 cm^−1^ (N–H bending, Amide II) [31,32], as well as at 1718–1722 cm^−1^, which correspond to the C=O carbonyl of the butyl acrylate ester, thus confirming the presence of butyl acrylate (BA) units [31]. Besides the specific peaks corresponding to hydrophilic amides bonds specified earlier, the bands assigned to C–H deformation modes are observable between 1350 and 1450 cm^−1^ (absorption bands at 1388 and 1368 cm^−1^) which are characteristic of the symmetric deformation of the –C(CH_3_)_2_ hydrophobic isopropyl group [25,33]. The all-data spectra of copolymer p(NIPAM-BA) show a peak at 1718–1722 cm^−1^, which corresponds to the C=O carbonyl of the butyl acrylate ester, thus confirming the presence of the hydrophobic butyl acrylate (BA) units [31]. In MAPLE samples (water, Figure 5A), for NH stretching vibrations occurring at 3301 cm^−1^, C (O), the carbonyl group C=O a butyl acrylate ester (BA) at 1718 cm^−1^ appears only for the drop-cast sample, (Amide I) at 1650 cm^−1^, and the bending vibration (Amide II) at 1540 cm^−1^; the appearance of the –C(CH_3_)_2_ hydrophobic isopropyl group for (350, 450 mJ/cm^2^) confirmed the structure of poly (N-isopropylacrylamide) [33]. When DMSO was used as solvent for the layers deposited by the MAPLE method at different laser fluences (Figure 5B), the carbonyl group C=O a butyl acrylate ester (BA) at 1718 cm^−1^ appears only for MAPLE samples only at a laser fluence of 350 mJ/cm^2^. In addition, these samples appear for the hydrophilic amides’ groups (Amide I and Amide II) for all coatings, as well the appearance of the hydrophobic isopropyl group in samples deposited at laser fluences of 350 mJ/cm^2^ and 450 mJ/cm^2^. In samples obtained with DMSO as a solvent, the S=O group at the 1044 cm^−1^ signal was observed, which is a peak that appears as the specific signature of the DMSO compound. A possible explanation for the presence of the S=O group signal/peak in p(NIPAM-BA)-based copolymer layers is the presence of a small amount of DMSO solvent that was not completely evaporated and absorbed outside the vacuum chamber during MAPLE deposition. Thus, in this case the best results were obtained at a laser fluence of 350 mJ/cm^2^ (DMSO, Figure 5B) where the C=O signal at 1718 cm^−1^ corresponding to BA is most pronounced.

Further investigations show that in the case of the copolymer layers obtained by the MAPLE method using chloroform, the peak corresponding to the BA component is observed at 1718 cm^−1^, corresponding to C=O (saturated ester) only for the drop-cast coating and at a laser fluence of 250 mJ/cm^2^, while the peaks observed at 3301 cm^−1^, 1649 cm^−1^, and 1543 cm^−1^ correspond to NH, Amide I, and Amide II (Figure 6C) for all samples while the hydrophilic isopropyl group disappeared. In addition, in the spin coating samples, the characteristic groups appear similar to the MAPLE deposition technique at 3301 cm^−1^ (NH) (Amide I and II) at 1649–1543 cm^−1^, and the bands assigned to C-H deformation modes are observable between 1350 and 1450 cm^−1^, which are characteristic of the symmetric deformation of the isopropyl group for all samples. The BA component corresponding to C=O (saturated ester) appears for the drop-cast and at a laser fluence of 250 mJ/cm^2^ (water and chloroform).

Overall, the conservation of the isopropyl hydrophobic groups along with the existence of the hydrophilic bonds, as evidenced in the above FTIR spectra, indicates the hydration/dehydration structure of the p(NIPAM-BA) coatings.

In the case of DMSO, the representative signals of the hydrophobic group, the C=O group of the BA component at the 1718 cm^−1^ signal, is the most pronounced.

### 2.4. Surface Composition Measurements by X-Ray Photoelectron Spectroscopy (XPS) Analysis

It is known that surface chemistry modification can be performed to decrease or increase protein/surface interactions [34]. Since such modification can occur by using different laser fluencies, the chemical surface composition changes of the p(NIPAM-BA) copolymer coatings obtained by drop-cast, MAPLE, and spin coating were further analyzed by XPS.

**Figure 7:**
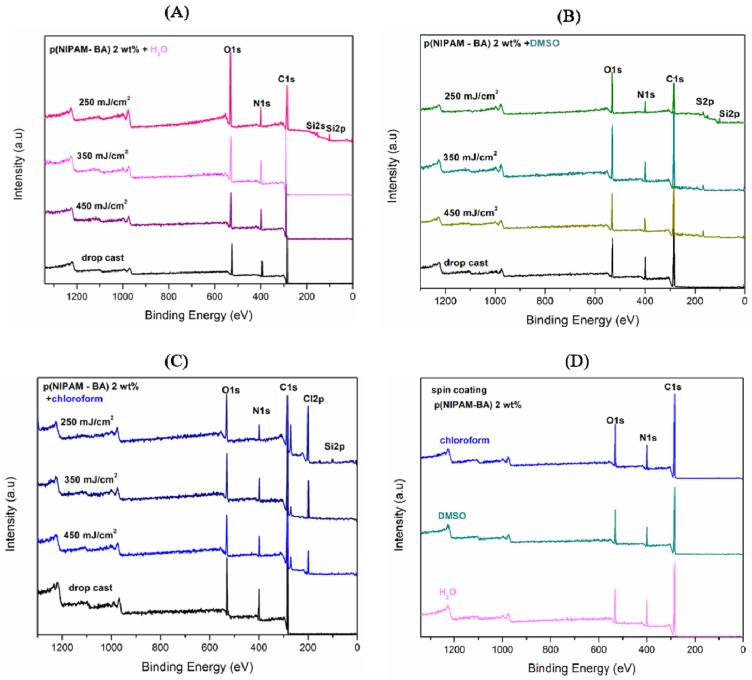
X-Ray photoelectron spectroscopy (XPS) wide survey spectra scan of p(NIPAM-BA) deposited by MAPLE using (**A**) water, (**B**) DMSO, and (**C**) chloroform, compared to drop-cast (black line) at varied laser fluences (250, 350, 450 mJ/cm^2^) (colored lines) and (**D**) spin coating at different solvents.

The XPS wide survey spectra obtained for MAPLE (Figure 7 A–C) and spin coating (Figure 7D) show typical signals associated with C1s (Carbon), N1s (Nitrogen), and O1s (Oxygen). The Si2p peak/signal was observed in all spectra of those deposited by MAPLE at the lowest laser fluence of 250 mJ/cm^2^, while in the case of the DMSO solvent used, the appearance of S2p peak (Figure 7B) and of Cl2p peak in the case of samples obtained when chloroform was used as solvent (Figure 7C) are observed. The p(NIPAM-BA) copolymer before (drop-cast) and after MAPLE deposition showed XPS peaks corresponding to C1s at 284.8, N1s at 399 eV, and O1s at 531.5–533 eV, in agreement with the elemental composition of the P(NIPAM-BA) coating. The XPS spectra show that all samples present a peak centered at 284.8 eV, corresponding to aliphatic carbons. Further, the O1s peak position at 531.5 eV for SC (i.e., C-O group), and at 533 eV (i.e., C=O) for MAPLE one, is consistent with the presence of organic matter in the surface of the copolymeric layer containing the C=O groups of p(NIPAM) [33].

Furthermore, the existence of oxygen (O1s) electrons detected on the surfaces of samples confirms that butyl acrylate (BA) hydrophobic structural units exist on the investigated samples’ surface [35].

The peak/signal that appears at 399.1 eV was attributed to the N1s binding energy of the hydrophilic amide group in the p(NIPAM) structural chain [36].

In the case of the coatings obtained by MAPLE at a laser fluence of 250 mJ/cm^2^ (Figure 7A), with water used as solvent, the presence of Si–O–Si on the surface [37] was given by the two peaks at 100.1 and 150.5 eV (assigned as Si2p and Si2s peaks to silicon (Si) element). This is an indication that the Si substrate used was not fully covered. The lack of Si2p peak in all the other types of depositions is an indicator for a homogeneous coverage of the Si substrate with P(NIPAM-BA) (i.e., C1s intensity comparable in different areas after MAPLE and spin coating).

The low-intensity peak at 164.0 eV, corresponding to the (Sp^2^) Sulphur element, was also detected in all MAPLE samples, Figure 7B, when DMSO was used as a solvent, detecting sulfur Sp^2^ as a fingerprint of the DMSO solvent. This is in agreement with FTIR investigations (Figure 5B).

No significant differences of the XPS measurements were observed for the samples deposited by MAPLE and spin coating of the p(NIPAM-BA), except that, in the case of the MAPLE method, with increasing laser fluence, the signals of S2p (Sulphur—Figure 7B) and Cl2p (Chlorine—Figure 7C) were much smaller, thus confirming a partial removal of the DMSO and chloroform solvents from the samples.

The results of XPS analysis for the elements’ atomic percentage of the copolymer surfaces before drop-cast and after deposition by MAPLE and SC are summarized in Table 2, giving an indication that neither the initial copolymer reaction with the DMSO or chloroform solvent nor the deposition techniques (MAPLE and spin coating) are involved in surface composition modification. Therefore, they are adequate to obtain homogenous copolymeric coatings.

The contribution related to C1s values decreases in all the coatings obtained by MAPLE regardless of the fluences used, but this is more pronounced in the case of using the lowest fluence, suggesting that the oxidation effect is occurring. An opposite behavior is observed for the O1s signal, increasing in the case of the coatings obtained by MAPLE, regardless of the used fluences, and the most important contribution being in the case of the lowest fluence. The moderate increase and differences are observed in the case of coatings obtained when using chloroform solvent and MAPLE, compared with the drop-cast or spin-coated coatings. This behavior suggests additional bonding with oxygen that appears on the copolymeric surface, suggesting an oxidation effect occurring in the ambient surrounding.

### 2.5. Contact Angles Measurements and Swelling Behavior

Surface wettability is one of the essential characteristics playing a role in cell attachment and protein adsorption/desorption at the surfaces of biomedical devices [38]. The contact angle is more susceptible to surface roughness, surface heterogeneity, and swelling behavior, as Tang et al. [39] described.

In our case, the water contact angle measurements performed at room temperature (RT 24 °C) and 37 °C (cell culture temperature), respectively, correspond to the cell culture conditions presented in Table 3, Table 4, Table 5 and Table 6.

The measurements performed at RT indicate a hydrophilic character for all the surfaces obtained by MAPLE with polar solvents (water—Table 3 and DMSO—Table 4), while for those obtained with the non-polar solvent (chloroform-Table 5), the hydrophobic character of surfaces obtained for the lowest fluence (250 mJ/cm^2^) changed to moderate hydrophilic with increasing fluence. The values indicating hydrophobicity were observed as well in the case of the coatings obtained by chloroform by drop-casting.

Despite the increase in contact angle values with increasing fluence, due to surface topography changes, which were also observed previously in the case of polymeric coatings obtained by MAPLE [26], the surfaces in the present study still remain hydrophilic. Similar values for the CA in the case of the coatings obtained with DMSO (350 mJ/cm^2^) and chloroform (350–450 mJ/cm^2^) were reported by Gilcreest et al. (contact angle for PNIPAM (74.5 ± 0.2 degrees) and for copolymers of N-isopropyl acrylamide with N-tert-butyl acrylamide (NtBA)) [40].

There were small increases observed in CA values when the temperature was shifted from RT to 37 °C for the coatings obtained by MAPLE when using water as the solvent. On the contrary, for the coatings obtained with DMSO and chloroform in the interval 350–450 mJ/cm^2^, the values slightly decreased for the measurements performed at 37°, indicating that hydrating behavior occurred. This was observed especially for the coatings obtained with DMSO at 450 mJ/cm^2^, when a decrease from 84° to 61° took place. This behavior could be explained by the presence of DMSO in the coatings, as shown by FTIR and XPS, as the formation of miscible DMSO-H_2_O clusters could be formed, as reported by Zohuriaan et. al. [41]. The above observed variations could also be explained by the fact that it is generally accepted that contact angle measurements are commonly applied to analyze the surface wettability of materials, but they are generally not useful for porous surfaces owing to the heterogeneity, capillary force in pores, and surface reconstruction [36]. However, comparing poriferous samples with temperature changes can give an idea of surface behavior on the temperature switch.

The exception observed in the case of the coatings obtained with chloroform at the lowest fluence could be related to the fact that the coating is thinner, without the presence of grouped clusters on its surface, that could interfere with its ability to expand/fold.

The CA values measured for layers deposited with SC (Table 6) did not present significant differences below or above polymer transition temperature for water (an increase of fewer than two degrees) and chloroform solvents (a decrease of three degrees), except for DMSO, when a transition from 59.2° to 66.78° was observed when measuring at 37 °C.

The hydration effect observed for most cases can be explained by the network of hydrogen bonded water molecules at the copolymeric interface, with a coil-to-globule transition related to a significant rearrangement of the solvent in the proximity of the surface of the polymer [42].

These observations were further confirmed by immersing the copolymeric coating in water at room temperature.

**Figure 8:**
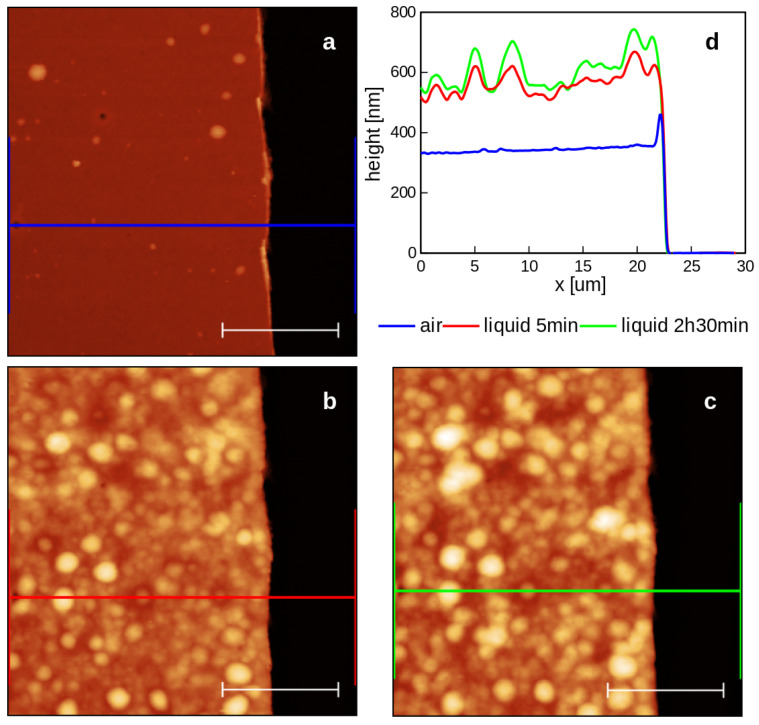
Surface topography in the vicinity of a step created on the p(NIPAM-BA) coating deposited by MAPLE with DMSO solvent, at a laser fluence of 350 mJ/cm^2^. The same area of the sample was imaged in air (**a**) and in water, a few minutes after immersion (**b**) and roughly 2 h and 30 min after immersion (**c**). For a clearer visualization of the evolution of the coating’s thickness upon immersion in water, each profile shown in (**d**) is an average of 128 parallel profiles from the areas delimited by the end markers of the colored segments shown in the AFM images. The lateral scale bars are 10 µm. The color palettes of the AFM images are matched.

The effects of water on the deposited p(NIPAM-BA) were also studied by imaging the topography of the same area in ambient air and subsequently in water at room temperature. In order to allow the measurement of the layer’s thickness in air and in water, part of the deposited material had been scraped with a cutter blade to create a step. It can be observed that both morphology and thickness are subject to major changes. The thickness values (averaged over the entire scanned areas) are: 347 nm in air; 570 nm in water after a few minutes of immersion; and 611 nm after roughly 2 h and 30 min of immersion. The morphology changes dramatically, with local swelling of the material creating globular-like structures. Some pores remain visible on the surface of the hydrated material despite the thickness increase of more than 150% compared with the initial layer thickness (Figure 8).

### 2.6. Biological In Vitro Investigations

#### 2.6.1. Cell Viability

The mechanical and physical–chemical characteristics of the biomaterial surface (i.e., topography, roughness, hydrophilicity) influence cell biology, and thus cell–biomaterial interactions are important for the evaluation and improvement of the efficacy of material for further in vivo studies [43,44].

In this work, we investigated the differences in surfaces properties of p(NIPAM-BA) layers obtained by MAPLE and SC deposition methods. Using H_2_O/DMSO chloroform as a solvent can modulate cellular response in vitro. Whether the response may vary depending on the cell type was also evaluated. Since one of the medical applications of p(NIPAM-BA) based coatings is their use as an efficient antitumoral drug delivery system, the effect of p(NIPAM-BA) layers’ surfaces on the viability and proliferation of normal and tumoral cells was evaluated. Two cell lines of different origins were used in our experiment, human embryonic kidney HEK 239T cells, commonly used as a normal cells model, and murine melanoma B16-F1 cells. Cell toxicity induced by different coatings was investigated at 24 and 48 h and compared to the control (glass) using MTS colorimetric assay. The coverslip was used as a supplementary cell proliferation assay control.

**Figure 9:**
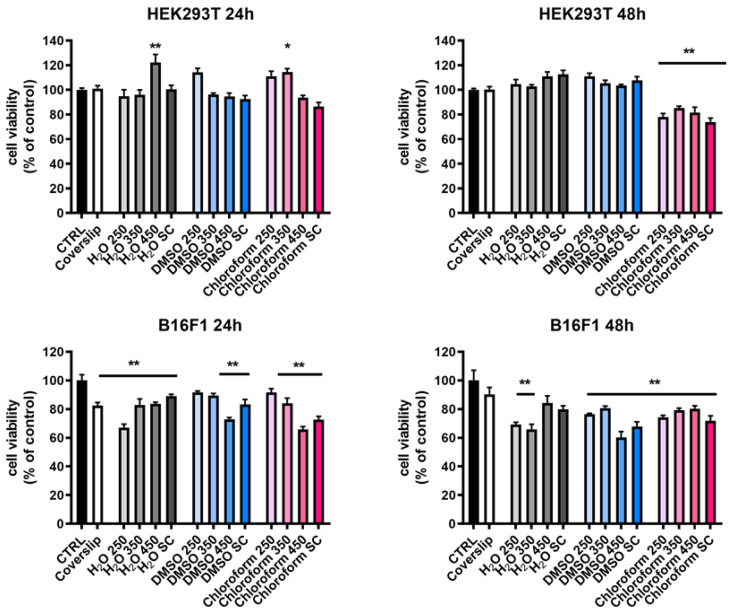
Viability of HEK 293T and B16-F1 cells grown on different biomaterials for 24 and 48 h was determined by MTS assay. The values are expressed as % of control mean ± standard deviation values (*n* = 6); * *p* < 0.05 and ** *p* < 0.01 vs. CTRL (glass). The fluences are expressed as mJ/cm^2^.

Experiments conducted with both cell lines have shown that viability and proliferation are dependent on the cell line and deposition method of the polymeric coatings. The coverslip supplementary control revealed good proliferation for both the cell lines and time points indicated. The results presented in Figure 9 reveal a minimal impact of different tested biomaterials on normal HEK 293 T cells after 24 h of incubation. However, a significant increase in cell viability for MAPLE with H_2_O (450 mJ/cm^2^) (120%, ** *p* < 0.01 vs. CTRL, glass) and chloroform (110%, * *p* < 0.05 vs. CTRL) was observed. At 48 h, cells grown on biomaterials doubled the proliferation rate as observed from OD values, which are directly proportional to the number of living cells (Supplementary Figure S2). The calculated cell viability values (%) were similar to the control, the exception being observed for the cells cultured on the layers obtained by MAPLE (all fluences) and SC deposition, using chloroform as solvent. In these cases, a slight decrease in cell number to approximately 80% was observed (** *p* < 0.01 vs. CTRL). It is worth noting that this level can be considered non-toxic or weak toxic in accordance with ISO legislation [45]. A non-toxic effect on HEK cells and mouse fibroblasts induced by pNIPAM and poly(N-isopropylacrylamide-co-butylacrylate) hydrogels was reported by Capella V et al. [46] and Becerra N et al. [13].

Unlike normal cells, a time-, method-, and solvent-dependent cytotoxic effect was found for murine melanoma B16-F1 cells. Thus, after 24 h the coatings were non-toxic according to ISO legislation (>82% viability), a significant decrease in cell viability compared to control (** *p* < 0.01 vs. CTRL) being obtained for H_2_O 250 mJ/cm^2^, (~65%), DMSO 450 mJ/cm^2^, (~70%), and chloroform (SC and 450 mJ/cm^2^ (~65–70%). After 48 h of incubation, cancer cells also proliferate also but not as cells cultured on the control (Supplementary Figure S2). Increasing the time of cell-surface contact to 48 h resulted in a statistical reduction of the proliferated rate and viability of cells grown on all the layers (Figure 9 and Supplementary Figure S2), irrespective of the method and solvent used (60–78% cell viability; ** *p* < 0.01 vs. CTRL). The exception is MAPLE H_2_O 450 mJ/cm^2^ coating (>80% cell viability).

#### 2.6.2. Adhesion and Distribution of Cells onto the p(NIPAM-BA) Layers

**Figure 10:**
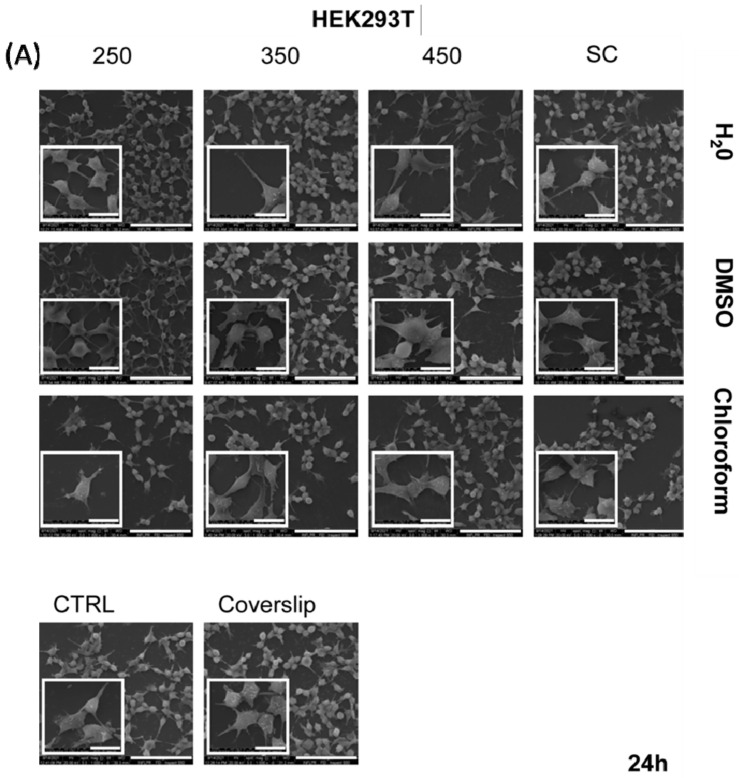

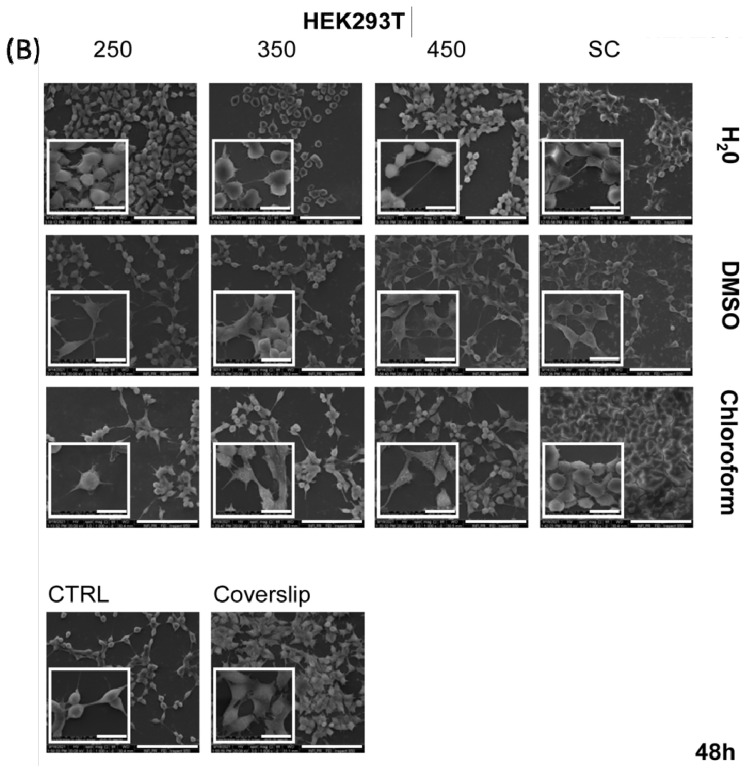
Adhesion and distribution of normal HEK293T cells cultured on p(NIPAM-BA) copolymer coatings for (**A**) 24 and (**B**) 48 h. Representative images (1000×; inserts 5000×) of the attached cells obtained by SEM microscopy. Scale bar 20μm. The fluences are expressed as mJ/cm^2^.

**Figure 11:**
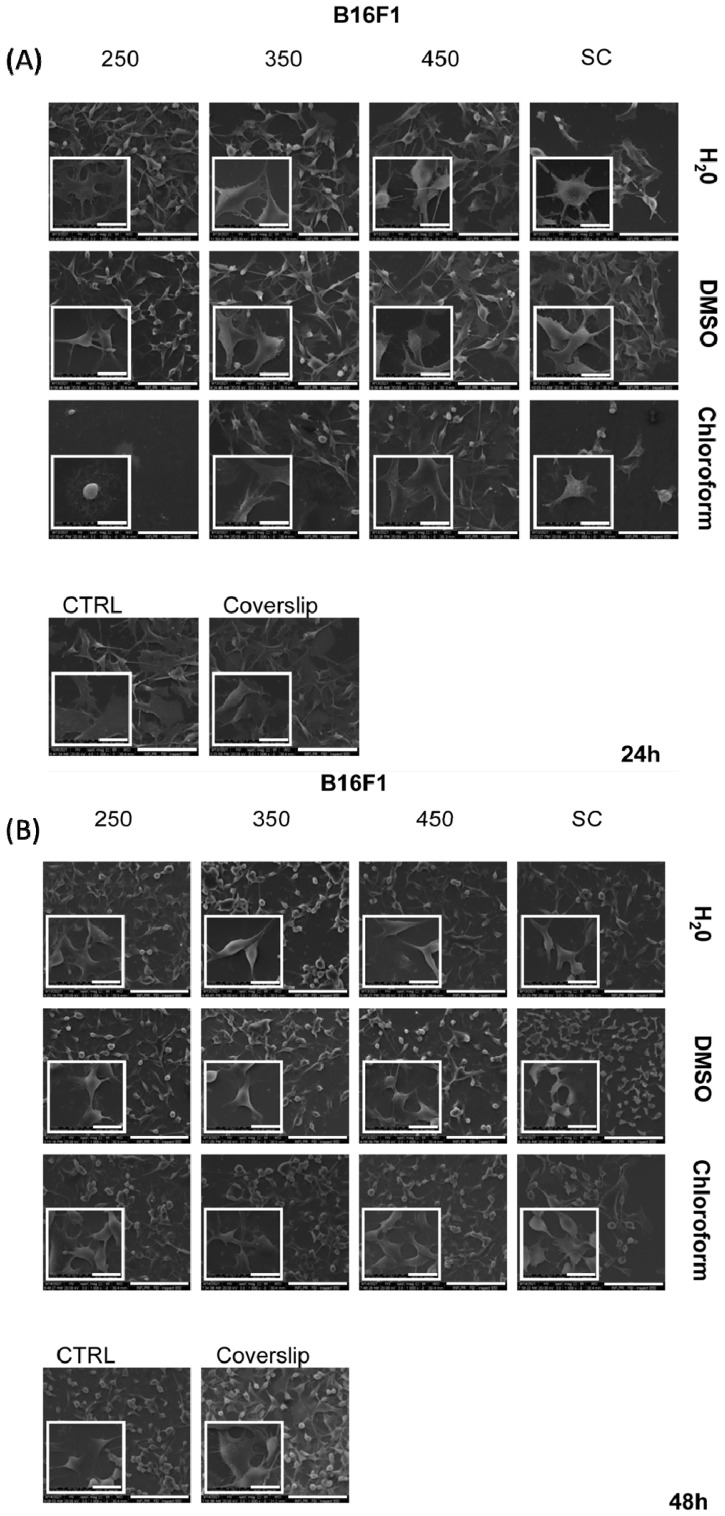
Adhesion and distribution of tumoral B16F1 cells cultured on p(NIPAM-BA) copolymer coatings for 24 h (**A**) and 48 h (**B**). Representative images (1000×; inserts 5000×) of the attached cells obtained by SEM microscopy. Scale bar 20 μm. The fluences are expressed as mJ/cm^2^.

Cell morphology and cells interaction with p(NIPAM-BA) copolymer coatings are important aspects to evaluate to understand the influence of biointerface properties on cell behavior. To observe whether cell morphology and behavior varied as a function of surface properties, HEK 293T and B16F1 cells were examined by scanning electron microscopy after 24 and 48 h of culture in direct contact with the coatings. The results obtained in the cytotoxicity experiment are in agreement with the morphological analysis performed by SEM (Figure 10 and Figure 11).

The HEK293T cell presented normal epithelial cell character and a small polygonal shape on all surfaces, as well as on the control after 24 h of culture (Figure 10A). A uniform distribution of adhered cells on all copolymer surface samples was observed. Moreover, a relatively flat morphology as well as short pseudopodia extensions of the cell membrane were detected on all coatings tested, resembling the control at 24 h. After 48 h, an increasing number of adhered normal HEK293T cells were observed in patches on biointerfaces, covering a larger area of the surface (Figure 10B).

This observation suggests that clustered cells at 48 h have originated and proliferated highly from strongly adhered cells at 24 h incubation on copolymer surfaces. These data correlate with the results obtained in the proliferation assay after 48 h cultivation on different copolymer surfaces (Supplementary Figure S2). In addition, cell morphology was preserved and also a stronger cell–cell and cell–substrate interaction could be seen as a good indicator of retained adhesion on the p(NIPAM-BA) copolymer coatings after 48h. However, an increased number of HEK293T cells that adopted a rounder morphology can be observed in the samples which contain p(NIPAM-BA) layers obtained by MAPLE deposition that used chloroform solvent (insert images, Figure 10B). This behavior might be due to the trace of chloroform within the coatings, as confirmed by FTIR and XPS.

B16F1 melanoma cells adhered strongly to all copolymer surfaces as well as on the CTRL and coverslip control (Figure 11A,B). The melanoma cells adopted flattened morphology as a monolayer surface, widespread onto the polymeric substrates. Although cells exhibited their native shape with a large cytoplasm area and numerous filopods, the samples with p(NIPAM-BA) layers significantly reduced the proliferation and number of murine melanoma cells compared to CTRL, at both time points. After 48 h of culture, the cell area was reduced and cell shrinkage and a modified cellular shape to a spindle-like morphology were detected in all p(NIPAM-BA) layers tested (Figure 11B). As for HEK cells, these observations on melanoma cells’ SEM images correlate with the results from the proliferation assay (Supplementary Figure S2). Cell morphology is important in the regulation of cell activities. Maintaining native cell morphological characteristics is critical to maintaining cancer cell characteristics. Altered cancer cell morphological characteristics observed in our study could be an indicator of the anti-tumoral capacity of p(NIPAM-BA) layers.

To enhance the desired cellular response, several characteristics of the biomaterial’s surface such as hydrophobicity/hydrophilicity, chemistry, roughness, etc. should be considered. The pNIPAM copolymer can influence cell adhesion, which is essential in pathological processes such as cancer [47]. It was reported that the hydrophobicity of pNIPAM could affect the adhesion of cells of different origins due to its capacity to adsorb matrix proteins (i.e., fibronectin) and growth factors stimulating cell growth [48]. The dependence of cell behavior on the type of cell line used in biological experiments evaluating pNIPAM copolymer was also reported by others [33,49,50].

In our study, all the p(NIPAM-BA) coatings showed hydrophilic surfaces, with the lowest hydrophilicity exhibited by the layers obtained by MAPLE and SC deposition procedures and chloroform solvent (Figure 8), where a significant reduction in cell viability was observed, especially at 48 h for both cell lines (Figure 9A,B). Roughness also influences cell behavior; cells such as fibroblasts prefer smooth surfaces, while osteoblasts adhere better on coatings with higher roughness [18,50,51,52,53,54]. In our case, MAPLE layers using DMSO exhibit the lowest roughness compared to H_2_O and chloroform and together with their hydrophilic character conducted a better survival of normal cells compared to melanoma cells. All these properties support their application as good delivery vehicles for anti-tumoral agents.

## 3. Materials and Methods

### 3.1. Materials and Reagents

P(NIPAM-BA) copolymer (C_6_H_11_NO)m(C_7_H_12_O_2_)n (butyl acrylate 5 mol %, average Mn 30.000, LCST of 25 °C) and dimethyl sulfoxide (DMSO solvent, ACS reagent, ≥99.8) were purchased from Merck (Sigma-Aldrich, Spruce Street, 3050, Saint Louis, MO 63103, USA). Chloroform (EMSURE^®^ ACS, ISO, Reag. ≥99.8%, Ph Eur) was purchased from (SUPELCO, Darmstadt, Germany). All the reagents were used without further purification.

### 3.2. Surface Designing/Tailoring/Deposition

#### 3.2.1. MAPLE Method

MAPLE method consists in using a laser beam for evaporating a frozen material (target) within a vacuum chamber and collecting the material onto a support placed parallel and in target proximity (3–5 cm). The setup used for obtaining the copolymeric coatings was described in [55]. In this work, a Nd: YAG pulsed laser system (SURELITE II™, Continuum Company, Pessac, France) (working at 266 nm wavelength, 10 Hz repetition rate, and 6 ns pulse duration) was used for deposition of the p(NIPAM-BA) coatings. The UV pulsed laser beam was directed onto the cryogenic target located in the vacuum deposition chamber through a lens with a focal length of 75 mm. The target consisted in p(NIPAM-BA) copolymer dissolved (2 wt.%) in three different solvents: Milli-Q ultrapure water, dimethyl sulfoxide (DMSO), and chloroform (CHCl_3_) and frozen by liquid nitrogen. A cooling system and a copper target support were used to maintain the solutions frozen during experiments. The fluences used were: 250, 350, and 450 mJ/cm^2^. The number of pulses was maintained at 72k pulses for water and DMSO, while for chloroform, due to its high evaporation rate, the number was reduced to 36k pulses to obtain coatings in the same thickness range. The obtained values for thicknesses for MAPLE samples were 179–190 nm for the lowest fluence (179 nm water, 181 DMSO, and 191 nm chloroform) and for medium fluence varied from 211 nm for water and 217 DMSO to 345 nm for chloroform, and for 450 mJ/cm^2^, the thicknesses varied from 250 in the case of water to 600 nm in the case of chloroform. The substrate was positioned parallel to the target at a fixed distance (3.5 cm), and during p(NIPAM-BA) deposition, the volatile molecules of the solvent were removed by means of vacuum pumps. The background pressures (air) ranged from 3 to 4 × 10^−3^ Pa, as obtained with a Pfeiffer-Balzers TPU 170 turbomolecular pump (Albuquerque, NM, USA). For each condition, at least three samples per type of analysis were obtained, except for biological studies, where triplicates and repetition were necessary. In order to avoid the variation of sample characteristics from batch to batch, targets were prepared using the same volume and the fluences and pressures were monitored during the depositions. Nevertheless, at high fluences in the case of chloroform solvent, due to higher evaporation rate, some variations on surface morphology might appear.

#### 3.2.2. Spin Coating Method

Laurell Tech spin coater (Laurell Technologies, North Wales, PA, USA) was used to prepare the p(NIPAM-BA) layers by spin coating method, chosen to assure similar medium thicknesses (300 nm) to those used in biological assays. The spin coating technique is widely used for the fast production of polymeric coatings, where a centrifugal force results in a uniform spread using a small amount of the copolymer and making possible deposition on a larger surface of the substrate. The main advantages of the technique depend on dispersion volume and rotational parameters that are sensitive to copolymeric solution properties. In the case of using water and chloroform as solvents, the coatings were obtained in a single step of 4500 rotation per minute for 20 s, while in the case of DMSO, two steps were used: 5000 rpm for 90 s, followed by 6500 rpm for 50 s.

### 3.3. p(NIPAM-BA) Morphologic Analysis

#### 3.3.1. Scanning Electron Microscopy (SEM)

Topographical investigations of the p(NIPAM-BA) surfaces were done by means of (SEM) with a JSM-531 Inspect S. system (Hillsboro, OR, USA) at an accelerating voltage of 20 kV. The layers were air-dried and covered using a sputtering coater with 10 nm Au (Agar Scientific Ltd., Essex, UK).

#### 3.3.2. Atomic Force Microscopy (AFM)

The surface morphology and the overall roughness (quantified by the root mean square roughness) of the p(NIPAM-BA) layers were analyzed by AFM (XE 100, Park Systems, Suwon, South Korea). Imaging was performed in non-contact mode, using silicon tips in ambient conditions. In the case of measuring the hydration of the coatings, imaging was performed in ambient conditions in air and in liquid (aqueous solution) in non-contact mode, using the same AFM tip (AC160TS: Olympus Europa, Hamburg, Germany) and without changing the lateral position of the sample. The roughness values were measured in different areas of the samples and for at least three samples; the values were mediated.

### 3.4. Wettability Characterization by Contact Angle (CA)

The wetting behavior was achieved by measuring the surface contact angle (CA) on the p(NIPAM-BA) layers by using the classic sessile drop method applied for two different temperatures (24 °C and 37 °C). CA measurements were conducted using a KSV CAM101 microscope (KSV Instruments Ltd., Espoo, Finland) system equipped with a video camera and FireWire interface (resolution of 640 × 480 pixels). In all the cases, droplets of 3 μL were positioned on the p(NIPAM-BA) surface on different locations of the sample’s surface, and the values were mediated (3 measurements per sample, three samples).

### 3.5. Chemical Profile: Analysis of p(NIPAM-BA)

#### 3.5.1. Fourier Transform Infrared Spectroscopy (FTIR) Analysis

The specific chemical structure of the p(NIPAM-BA) coatings obtained by MAPLE and spin coating was investigated by Fourier Transform Infrared Spectroscopy (FTIR) method using a JASCO Inc. 28600 (Mary’s Court, Easton, MD 21601, USA). Drop-casted material was used as a reference to determine the main characteristic IR vibrations of functional groups of the p(NIPAM-BA). All the samples were measured in the 400–7800 cm^−1^ range, with a resolution of 4 cm^−1^, in absorption mode by the accumulation of 1024 scans, while those for drop-cast were measured in ATR mode (Attenuated Total Reflectance). The absorbance was normalized, and baseline correction was applied.

#### 3.5.2. X-ray Photoelectron Spectroscopy (XPS) Analysis

To study the surface chemistry of layers, an Escalab Xi+ system (Thermo Scientific, Waltham, MA, USA) was used for X-ray photoelectron spectroscopy (XPS) survey and high-resolution XPS spectra acquisition. All the survey scans were acquired using an Al Kα gun with a spot size of 900 µm and a pass energy of 50.0 eV, with an energy step size of 1.00 eV (5 scans). The excessive charging load of the layers has been reduced with the help of a flood gun. In the case of the high-resolution XPS spectra data, the pass energy used was set to 10.0 eV, and the energy step size was 0.10 eV with 15 scans that were accumulated for O1s, 10 for N1s, and 5 for O1s.

### 3.6. Biological Investigations

The p(NIPAM-BA) coatings were air-dried and covered with 10 nm Au before microscopy characterization using a sputtering coater (Agar Scientific Ltd., Essex, UK).

Prior to in vitro evaluation, all materials were sterilized for 30 min in antibiotics (penicillin 10.000 units/mL, streptomycin 10.000 μg/mL, Gibco by Life Technologies, Thermo Fisher Scientific, Waltham, MA, USA) in 1% phosphate-buffered saline (PBS) solution to prevent microbial contamination.

#### 3.6.1. Cells

Human epithelial embryonic kidney HEK 293T cells (ATCC, CRL-1573, Manassas, VA, USA) were cultured in Dulbecco’s Minimal Essential Medium (DMEM) medium (Gibco, Life Technologies, NY, USA), supplemented with 10% fetal calf serum (FCS) and 1% penicillin/streptomycin (all Gibco, Life Technologies, NY, USA) and maintained at 37 °C with 5% CO_2_.

Murine melanoma B16-F1 cells (ECACC, Porton Down, UK) were cultured in RPMI 1640 medium (Gibco, Life Technologies, NY, USA) containing glutamine and supplemented with 10% inactivated fetal calf serum (FCS (*v*/*v*), 1% penicillin/streptomycin and maintained at 37 °C with 5% CO_2_).

Cell viability was monitored using 0.4% trypan blue dye staining (Invitrogen, Thermo Fischer Scientific, Life Technologies Co, Eugen, OR, USA) and only cells with more than 90% viability were used for biological study. All experiments involving HEK 293T and B16-F1cells were performed in triplicate, twice, *n* = 6.

#### 3.6.2. Cell Viability Assay

Viability of HEK 293T and B16-F1cells grown on biomaterials and coverslip (CS) was evaluated by MTS ([3-(4,5-dimethylthiazol-2-yl)-5-(3-carboxymethoxyphenyl)-2-(4-sulfophenyl)-2H-tetrazolium, inner salt]) assay (CellTiter 96^®^ Aqueous Non-Radioactive Cell Proliferation Assay, Promega, Fitchburg, WI, USA) according to the manufacturer’s instructions. This quantitative colorimetric method is based on the conversion of the tetrazolium compound to a formazan dye by mitochondrial dehydrogenases from viable cells.

HEK 293T and B16-F1cells were seeded onto biomaterials and coverslip CS at an initial density of 50.000 cells/well for both cell lines and after 24 or 48 h of incubation in a humidified atmosphere (5% CO_2_ at 37 °C), the materials were rinsed three times with PBS to remove non-adherent cells. To determine cell metabolic activity, a mix of MTS reagent and cell culture media was added to each well. After 10–15 min of incubation, 100 μL of the culture solution was transferred to a 96-well clear bottom plate (Nunc, Thermo Fisher Scientific, CA, USA) and optical density was measured by a microplate reader (Mithras LB 940 Berthold Technology, Bad Wildbad, Germany) at 450 nm.

#### 3.6.3. Cell Adhesion by Scanning Electron Microscopy (SEM)

HEK 293T and B16-F1cells grown on biomaterials for the indicated intervals were washed with PBS and fixed with 2.5% glutaraldehyde in PBS for 20 min. Then, the samples were subjected for gradient dehydration and drying for 2 rounds of 15 min each with 50%, 70%, and 100% ethanol and 2 rounds of 3 min each with 50% and 75% hexamethyldisilazane (HMDS, Sigma-Aldrich, St. Louis, MO, USA) solution in ethanol and finally with 100% pure HDMS solution. Specimens were air-dried overnight in a chemical fume hood and metalized prior to scanning. The probes were employed for HMDS evaporation in a Bioair Euroclone AURA 2000 M.A.C., fume hood (Euroclone SpA, Siziano (PV), Italy). SEM imaging was conducted on a scanning electron microscope (Inspect S Electron Scanning Microscope, FEI Company, Hillsboro, OR, USA).

#### 3.6.4. Statistical Analysis

All values are expressed as mean ± SD. Data were analyzed with one way ANOVA, followed by Dunnett’s multiple comparisons using GraphPad Prism software for Windows version 5 (manufacturer, GraphPad Software, Inc., version 5; La-Jolla, CA, US). Statistical significance was set at *p* < 0.05.

### 3.7. Samples/Batch Production

In the case of MAPLE and spin coating, at least three samples per type of analysis were obtained and evaluated for each condition, while for biological studies 6 samples per type of assay and per type of analysis were obtained. No significant variation from batch to batch was observed in terms of FTIR and XPS. The variation in morphology was not significant.

## 4. Conclusions

The Matrix-Assisted Pulsed Laser Evaporation method was used for obtaining new synthetic biointerfaces of pNIPAM-BA, by combining the effect of laser fluence values with different solvents to obtain appropriate surface for cell–biointerface interaction. The spin coating method was used to obtain reference coatings. It was shown that solvents (chloroform, dimethyl sulfoxide, and water) as well as fluence variation (250–450 mJ/cm^2^) have an effect on the morphological, roughness, wettability, and chemical characteristics of the coatings.

Altogether, the AFM and SEM studies indicate smooth and similar surfaces obtained by spin coating, while in the case of MAPLE, a linear increase in roughness with increasing laser fluence was observed and, in combination with the use of solvents, surfaces characterized by nano-globular structures to pores presence were obtained.

The chemical functionality was kept for all the samples obtained by MAPLE and a tendency for hydration of the samples was observed in most cases, except those obtained for chloroform at 250 mJ/cm^2^.

The impact of surface characteristics of the p(NIPAM-BA) spin-coated and MAPLE layers on normal and tumoral cell viability and morphology were analyzed using colorimetric (MTS) and SEM. The viability of normal HEK 293 T cells was not affected after 24 or 48 h of cultivation on all the p(NIPAM-BA) layers compared to the control, regardless of the method and solvent used, except for the layers obtained using chloroform as a solvent. Nevertheless, the method and the solvent-dependent cytotoxic effect were found for murine melanoma B16-F1 cells, with a statistically significant reduction of the viability of cells grown on all the layers, irrespective of the method and solvent used.

Biological assays performed in vitro proved morphological and proliferation changes conditioned by the cell line and type of coating preparation.

Altogether, the physical-chemical and biological analysis revealed that the MAPLE deposition method using DMSO solvent and fluences of 350–450 mJ/cm^2^ is more appropriate for bioengineering applications due to the surface characteristics and the good compatibility with normal cells and cytotoxicity against melanoma cells.

## Figures and Tables

**Table 1:** Mediated values of p(NIPAM-BA) layers roughness’s obtained by MAPLE and spin coating technique.

		Roughness—RMS (nm)		
Deposition Methods		p(NIPAM-BA) 2 wt.%+ H_2_O	p(NIPAM-BA) 2 wt.%+ DMSO	p(NIPAM-BA) 2 wt.%+ Chloroform
**Drop-cast**		4.4	0.8	2.8
**MAPLE**	250 mJ/cm^2^	36.3	1.5	25.7
	350 mJ/cm^2^	139	27.7	143
	450 mJ/cm^2^	346	49.4	153.2
**Spin Coating**		3.6	7	0.9

**Table 2:** p(NIPAM-BA) atomic composition of drop-cast coatings deposited by MAPLE and spin coating.

Solvent Used	Deposition Technique					
	Drop-Cast	MAPLE Laser Fluence			Spin Coating	Atomic Composition (%)
		250 mJ/cm^2^	350 mJ/cm^2^	450 mJ/cm^2^		
**Water**	73.48	54.54	69.73	72.10	73.23	**C1s**
	12.01	9.66	13.19	13.18	13.03	**N1s**
	14.51	26.79	17.08	14.71	13.74	**O1s**
	-	9.01	-	-	-	**Si2p**
**DMSO**	73.72	55.96	64.57	64.00	72.99	**C1s**
	12.30	9.91	11.76	11.17	13.57	**N1s**
	13.98	27.34	20.30	20.77	13.44	**O1s**
	-	6.79	3.38	4.06	-	**S2p**
**Chlorofom**	72.71	59.83	67.83	66.16	74.15	**C1s**
	12.82	5.62	10.70	9.23	12.78	**N1s**
	14.47	18.04	16.63	17.11	13.07	**O1s**
	-	16.51	4.84	7.50	-	**Cl2p**

**Table 3:** Contact angle values on p(NIPAM-BA) coatings obtained by drop-casting and MAPLE, when water was used as solvent, at laser fluences 250, 350, and 450 mJ /cm^2^.

p(NIPAM-BA) _Water		
Laser Fluence (mJ/cm^2^)	CA (RT) CA (37 °C)	
Drop-Cast	63.73 ± 0.34	70.87 ± 0.72
250	25.85 ± 0.52	28.24 ± 0.5
350	28.99 ± 0.13	33.4 ± 0.22
450	36.75 ± 0.41	45.07 ± 0.37

**Table 4:** Contact angle values on p(NIPAM-BA) coatings obtained by drop-casting and MAPLE, when DMSO was used as solvent at laser fluences 250, 350, and 450 mJ /cm^2^.

p(NIPAM-BA) _DMSO		
Laser Fluence (mJ/cm^2^)	CA (RT) CA (37 °C)	
Drop-Cast	75.79 ± 1.24	60 ± 0.76
250	56.47 ± 0.84	62.31 ± 0.46
350	78.49 ± 0.58	75.7 ± 0.66
450	84.14 ± 1.36	61.64 ± 0.89

**Table 5:** Contact angle values of p(NIPAM-BA) coatings obtained by drop-casting and MAPLE when chloroform was used as solvent, at laser fluences 250, 350, and 450 mJ /cm^2^.

p(NIPAM-BA) _ Chloroform		
Laser Fluence (mJ/cm^2^)	CA (RT) CA (37 °C)	
Drop-Cast	92.58 ± 1.89	70.37 ± 0.79
250	91.09 ± 1.65	99.44 ± 1.53
350	77.44 ± 0.68	74.3 ± 0.85
450	77.89 ± 0.66	75.43 ± 0.98

**Table 6:** Contact angle values of p(NIPAM-BA) coatings when water, DMSO, and chloroform were used as solvents, obtained by spin coating.

p(NIPAM-BA)	CA (RT)	CA (37 °C)
**Water**	63.73 ± 0.99	65.36 ± 0.36
**DMSO**	59.2 ± 0.24	66.78 ± 0.15
**Chloroform**	66.77 ± 1.166 68.77 ± 1.16	69.18 ± 0.72

## Data Availability

Not applicable.

## Supplementary Materials

The following are available online at https://www.mdpi.com/article/10.3390/ijms23073988/s1.
