# Supplementary material for: New Poly(N-isopropylacrylamide-butylacrylate) Copolymer Biointerfaces and Their Characteristic Influence on Cell Behavior In Vitro
Source: Int J Mol Sci. 2022 Apr 3;23(7):3988. doi: 10.3390/ijms23073988 (PMC9000054; doi:10.3390/ijms23073988)
Supplement: Supplementary file 1 [file ijms-23-03988-s001.zip › ijms-1602579-supplementary.pdf]

Supplementary Data

New Poly(N-isopropylacrylamide-butylacrylate) Copolymer Biointerfaces and Their Characteristic Influence on Cell Behavior In Vitro

Nicoleta-Luminita Dumitrescu [1], Madalina Icriverzi [2], Anca Bonciu [3,4], Paula Florian [2], Antoniu Moldovan [1], Anca Roseanu [2], Laurentiu Rusen [1,*], Valentina Dinca [3,*] and Florin Grama [5,6]

1 Lasers Department, National Institute for Lasers, Plasma, and Radiation Physics, 409 Atomistilor Street, 077125 Magurele, Romania; nicoleta.dumitrescu@inflpr.ro (N.-L.D.); antoniu.moldovan@inflpr.ro (A.M.)
2 Ligand-Receptor Interactions Department, Institute of Biochemistry of the Romanian Academy, 060031 Bucharest, Romania; radu_mada@yahoo.co.uk (M.I.); florian_paula@yahoo.com (P.F.); roseanua@gmail.com (A.R.)
3 FOTOPLASMAT Department, National Institute for Lasers, Plasma, and Radiation Physics, 409 Atomistilor Street, 077125 Magurele, Romania; anca.bonciu@inflpr.ro
4 Faculty of Physics, University of Bucharest, 405 Atomistilor, 077125 Magurele, Romania
5 Department of General Surgery, Colțea Clinical Hospital, 1 I.C. Brătianu Street, 030171 Bucharest, Romania; florin.grama@umfcd.ro
6 Carol Davila University of Medicine & Pharmacy, 37 Dionisie Lupu Street, 030171 Bucharest, Romania
* Correspondence: laurentiu.rusen@inflpr.ro (L.R.); valentina.dinca@inflpr.ro (V.D.)

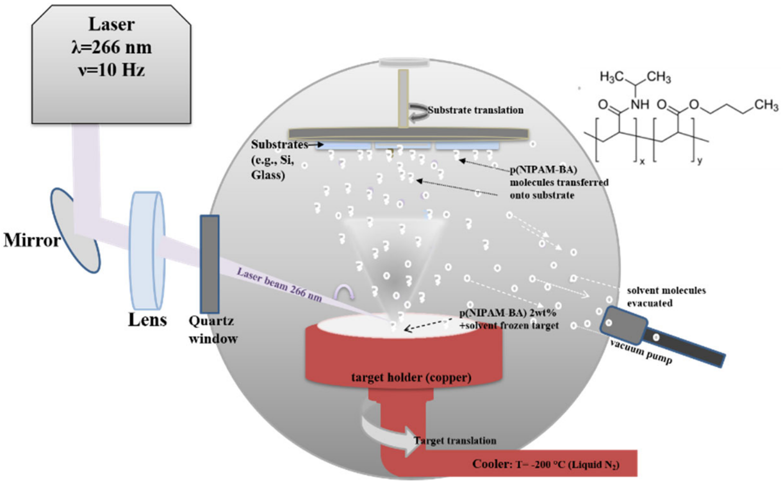

Figure S1. Experimental scheme of MAPLE method.

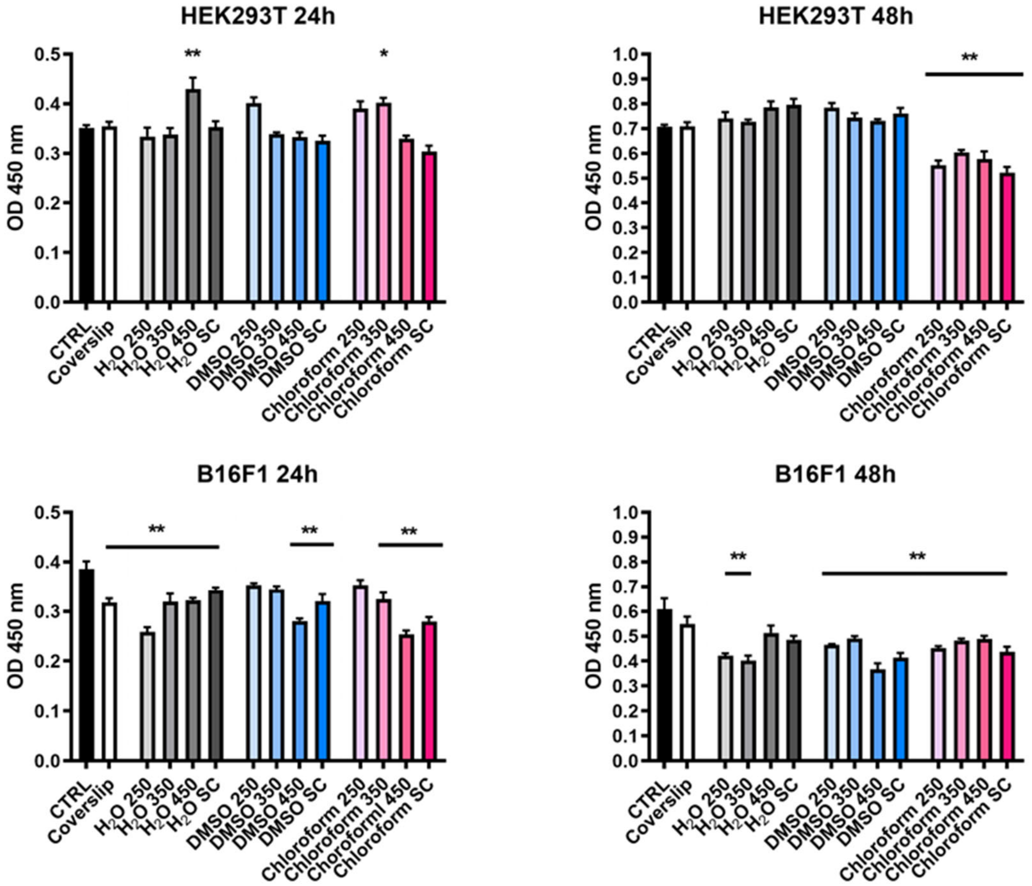

Figure S2. Viability of HEK 293T and B16-F1 cells grown on different biomaterials for 24 and 48 h was determined by MTS assay. The results are expressed as mean values of OD 450 ± standard deviation (n = 6); * $p < 0.05$ and ** $p < 0.01$ vs. CTRL (glass). The fluences were expressed as mJ/cm[2.]
